# The emotional well-being of Long COVID patients in relation to their symptoms, social support and stigmatization in social and health services: a qualitative study

**DOI:** 10.1186/s12888-022-04497-8

**Published:** 2023-01-25

**Authors:** M. Samper-Pardo, B. Oliván-Blázquez, R. Magallón-Botaya, F. Méndez-López, C. Bartolomé-Moreno, S. León-Herrera

**Affiliations:** 1 Institute for Health Research Aragon (IISAragon), Zaragoza, Spain; 2 Department of Psychology and Sociology, University of Zaragoza, Zaragoza, Spain; 3 Network for Research on Chronicity, Primary Care, and Health Promotion (RICAPPS), Barcelona, Spain; 4 Department of Medicine, University of Zaragoza, Zaragoza, Spain

**Keywords:** Long COVID, Emotional well-being, Social support, Stigmatization, Qualitative study

## Abstract

**Background:**

Long COVID patients have experienced a decline in their quality of life due to, in part but not wholly, its negative emotional impact. Some of the most prevalent mental health symptoms presented by long COVID patients are anxiety, depression, and sleep disorders. As such, the need has arisen to analyze the personal experiences of these patients to understand how they are managing their daily lives while dealing with the condition. The objective of this study is to increase understanding about the emotional well-being of people diagnosed with long COVID.

**Methods:**

A qualitative design was created and carried out using 35 patients, with 17 participants being interviewed individually and 18 of them taking part in two focus groups. The participating patients were recruited in November and December 2021 from Primary Health Care (PHC) centers in the city of Zaragoza (Northern Spain) and from the Association of Long COVID Patients in Aragon. The study topics were emotional well-being, social support networks, and experience of discrimination. All an inductive thematic content analyses were performed iteratively using NVivo software.

**Results:**

The Long COVID patients identified low levels of self-perceived well-being due to their persistent symptoms, as well as limitations in their daily lives that had been persistent for many months. Suicidal thoughts were also mentioned by several patients. They referred to anguish and anxiety about the future as well as a fear of reinfection or relapse and returning to work. Many of the participants reported that they have sought the help of a mental health professional. Most participants identified discriminatory situations in health care.

**Conclusions:**

It is necessary to continue researching the impact that Long COVID has had on mental health, as well as to provide Primary Health Care professionals with evidence that can guide the emotional treatment of these patients

## Background

The highly contagious coronavirus disease (COVID-19) that broke out at the end of 2019 led to a global pandemic [1, 2], posing a serious threat to health and well-being worldwide [3, 4]. The virulence of COVID-19 in the human body can vary greatly, with some patients displaying no symptoms and others dying from the disease [5]. It is estimated that around 10 to 20% of people affected by COVID-19 display ongoing symptoms for the months following the acute phase of the disease [6, 7]. In October 2021, the World Health Organization (WHO) released an official definition of the condition in the adult population, referring to it as a Post-COVID Condition [8]. This text will refer to it as Long COVID given its frequent use and wide acceptance in the scientific community.

### Long COVID

Long COVID is a multisystemic syndrome that is characterized by a variety of physical and neuropsychiatric symptoms that are persistent or cyclical and last for weeks after having contracted the acute Severe Acute Respiratory Syndrome Coronavirus 2 (SARS-CoV-2) infection [9–11]. Various studies indicate that Long COVID is more prevalent in adult women who have had severe COVID-19 symptoms and a history of psychiatric problems [5, 12, 13].

It is estimated that the average recovery period following an infection of COVID-19 varies between two to 3 weeks depending on the severity of symptoms [14–16]. The United Kingdom’s Office for National Statistics states that, irrespective of their severity, one in five people may experience symptoms after the first 5 weeks or more following the initial infection, while one in 10 people may experience symptoms after 12 weeks or more [17]. Due to this unprecedented scenario, at the end of 2020, the National Institute for Health and Care Excellence (NICE) announced that COVID-19 symptoms may last for four to 12 weeks, and thus diagnosing people who maintain or continue to develop symptoms that cannot be explained by an alternative diagnosis as having Long COVID [18]. In addition, proof of having COVID through the use of a diagnostic test was not set out as a criterion as many people did not take one, especially during the first months of the pandemic or in the case of asymptomatic patients [18].

Regarding the characteristic symptomatology of this new pathology, it is confusing, not very specific [19], and may be persistent or fluctuating over time [18]. Patients with Long COVID may present a combination of symptoms that have an effect at the respiratory, dermatological, cardiovascular, gastrointestinal, and/or neuropsychiatric levels [20–24]. The most predominant symptoms include extreme fatigue, shortness of breath, low-grade fever, cough, headache, chest and/or throat pain, muscle and joint pain, palpitations, diarrhea, loss of smell and/or taste, skin rashes, cognitive deficits such as mental fog, myalgias, and tingling in the upper and lower extremities [25–29]. Moreover, while less common, low oxygen saturations [30], as well as cardiovascular abnormalities such as arrhythmias, a high heart rate, myocarditis, or acute heart failure [31], have also been observed. It is important to note that similar symptoms, such as chronic fatigue, illness, or depression, were reported in patients during the SARS-CoV outbreak in 2002 [32].

The UK National Health Service (NHS) added depression and anxiety to the list of the most frequent COVID-19 symptoms, associating them as potential effects of experiencing extreme fatigue and other prolonged physical symptoms [33]. However, several studies point to the existence of neurological aspects that would contribute to this mental discomfort [12, 16].

### Long COVID and mental health

An increasing number of studies are researching the negative emotional impact on people who have had COVID-19 and have persistent symptoms; among the most prevalent mental pathologies are anxiety, depression, sleep disorders, post-traumatic stress disorder (PTSD), and mood fluctuations [34–39]. A recent report has detected similar symptoms in affected children [40]. The existing evidence suggests that patients with Long COVID have experienced reductions in their quality of life [41, 42]. A study carried out with hospitalized COVID-19 patients concluded that after eight to 12 weeks following the contraction of the infection, patients with persistent symptoms suffer a deterioration in all domains of their lives, including their mental health, when compared to the infected population without persistent symptoms [26]. In this respect, it has not yet been concluded whether these mental symptoms are triggered by the disease itself and its duration, or whether it is a neurological impact that causes them, such as a cognitive impairment caused by moving megakaryocytes from the bone marrow to the brain, thereby blocking blood flow [43]. In the case of psychiatric symptoms, COVID-19 could affect the brain indirectly by increasing cytokines [13], and some patients may even experience the appearance of white brain spots or microbleeds after the infection [44, 45]. Therefore, it is estimated that there may be a neurological cause that affects mental health after COVID-19 infection [12].

However, even setting aside the possible organic causes, genetic agents are not the only moderators of health, since there are various interactions between environmental and social factors on the health of the population. To our knowledge, the scientific evidence on the self-perceived mental health of Long COVID patients is still limited. Some qualitative studies have been identified where patients refer to their emotional well-being, in relation to their Long COVID pathology, highlighting the complexity and emotional challenges of living with the disease [46–51]. These patients are fearful about becoming reinfected with COVID-19 and experiencing the consequent deterioration and have anxiety due to the uncertainty regarding the evolution and scientific ignorance of society in general and, particularly, of health professionals [46, 48, 49]. Patients with Long COVID seem to be dissatisfied and disappointed with the treatment they receive from the health care system [50]. Likewise, some patients present depressive symptoms related to their own symptomatology and the physical state in which they find themselves compared to what is usual for them [49]. They state that they cannot recognize themselves, due to the significant changes they have experienced in terms of their capacities [51]. In addition, the change in sleep patterns, the inability to perform physical exercise, and a worse economic situation have been related to the emotional discomfort of these patients [52, 53].

Moreover, there are few studies on other social aspects, such as social support or society’s stigma and discrimination toward this group of patients. In general, people with adequate social support have a lower mortality risk compared to those without it [54]. Therefore, social isolation is considered a mortality risk factor for any cause of death [55]. This highlights the impact that poor social networks have on mental illnesses [56]. In general terms, social support can be a moderator for mental illnesses through other psychosocial factors [57, 58]. Various studies have examined social support through different lenses such as social integration and participation as well as both the real and perceived instrumental and emotional support received [59–61]. In relation to social isolation, there are other social variables such as stigma and discrimination. Consequently, some studies have delved into the stigma generated during the pandemic. Specifically, the study by Bhanot et al., (2021) indicates that the most stigmatized people have been those infected by COVID-19, the direct contacts of those affected, front-line health personnel, and people belonging to the lowest social classes. Among these groups would be Long COVID patients, as infected people and also with persistent symptoms, especially due to the fear of contagion generated at the beginning of the pandemic [62]. This stigmatization is in relation to the difficulty experienced in accessing different health services in the context of health care system deficits [63]. Consequently, this social rejection leads to the social isolation of these patients and negatively affects their physical and psychological health and general well-being. Furthermore, this discrimination may reduce their likelihood of seeking medical care and treatment, for fear of being shamed and stigmatized by society [62, 64].

Therefore, the objective of this study is to deepen our understanding of the emotional well-being of people diagnosed with Long COVID, as well as their social support and experiences of discrimination and social stigma.

## Methods

A qualitative design was created and carried out in order to collect information from patients suffering from Long COVID using an intentional sampling method. This research represents the first part of a study funded by the Carlos III Health Institute (PI21/01356), with the objective of establishing community interventions to improve the quality of life of patients with Long COVID using a citizen science approach.

Participants agreed to participate in the study and signed a consent form. Ethical approval was granted by the Ethics Committee for Clinical Research of Aragon (PI21/139 and PI21/454). All the procedures required for the development of this work complied with the ethical standards of this Committee and with the Declaration of Helsinki of 1975. All participants signed an informed consent form and their data were anonymized and only used for the purposes of the study.

In-depth interviews and focus groups were used to collect subjective data and gain an understanding of the processes involved in the generation of the discourse [65]. Highlighting the suitability of this type of methodology should be highlighted, since it allows us to delve into the subjectivity of the patient and generates significant evidence that brings us closer to understanding the emotional impact on various groups of people [66–68]. The research team used both individual interviews and focus groups to encourage more content to be shared in the discourse through social interactions. The study used two interviewers who were external researchers with scientific knowledge about Long COVID and who had previous experience in conducting qualitative research with Primary Health Care patients. Neither interviewer had had previous contact with any of the interviewees, nor were they aware of their identities. Before carrying out the interviews, they were both provided with the interview guides so that they could rehearse the individual interviews and focus groups through role-playing.

The participating patients were recruited from PHC centers in the city of Zaragoza (Northern Spain) and from the Association of Long COVID Patients in Aragon. When health professionals identified a potential patient, they informed them about the possibility of participating in the study and verified whether they met the inclusion criteria. Prior informed consent, as well as the individual’s data and contact details were provided to the research group so they could contact the patient and verify that they did not meet the exclusion criteria. Through this procedure, the members of the Association interested in participating were identified as potential candidates.

The inclusion criteria for participating patients were outlined as the following: 1) being over 18 years old; 2) having been diagnosed with Long COVID by a general practitioner (GP) or specialized doctor; and 3) having tested positive for COVID-19 (PCR, antigens test, or serology). The exclusion criteria were the following: 1) not being able to respond to the interviewer; 2) presenting a high cognitive impairment from any cause; 3) and be receiving palliative care.

Finally, 35 patients were included in the sample. An independent researcher carried out the individual randomization process using a computer-generated blind sequence with a list of participants. In total, 17 participants were interviewed individually and 18 participated in two focus groups. The allocation was not blind due to the nature of the study. A researcher called each participant to confirm the assigned intervention and their participation. The interviews and focus groups were carried out in November and December 2021. The data obtained in the study are considered representative of the population that met the inclusion criteria given that they are similar to other studies in terms of gender, age, number, and intensity of persistent symptoms [69–71].

All participants met the study criteria and agreed to participate in their assigned intervention. Table 1 shows the main demographic data of the 35 participants in terms of the variables: age, gender (men/women/other), marital status (single/married or in a relationship/separated or divorced/widowed), educational level (no formal studies but can read and write/primary education/secondary education/university education), employment status (employee/employee with temporary work disability/ unemployed, receiving unemployment benefits/ unemployed, not receiving unemployment benefits/ retired).

**Table 1:** Characteristics of participating patients

Variables	Patients (***n*** = 35) N (%)
Age	
20–40 years	8 (22.9%)
41–60 years	20 (57.1%)
> 60 years	7 (20%)
Gender	
Male	10 (28.6%)
Female	25 (71.4%)
Marital Status	
Single	4 (11.4%)
Married or in a relationship	19 (54.3%)
Separated or divorced	10 (28.6%)
Widowed	2 (5.7%)
Educational level (%)	
No formal studies but can read and write	1 (2.9%)
Primary education	3 (8.6%)
Secondary education	19 (54.3%)
University education	12 (34.3%)
Employment status (%)	
Employee	6 (17.1%)
Employee with TWD^a^	22 (62.9%)
Unemployed, receiving benefits	1 (2.9%)
Unemployed, not receiving benefits	1 (2.9%)
Retired	5 (14.3%)

^a^ TWD: temporary work disability

The sample included patients with various profiles in terms of their gender, age, time elapsed since infection, educational level, and marital status. Of the 35 patients, 71.4% were women, with a mean age of 49 years (SD: 10.81), with secondary education (54.3%) or university education (34.3%), married or in a relationship (54.3%), and in a situation of temporary work disability (62.9%). In addition, the average time elapsed since infection was 15 months (SD 4.0).

A standardized protocol was designed to guide the individual interviews and focus groups and was based on a list of topics and informal interviews with patients with Long COVID in the PHC setting that had been developed by other researchers who were developing other research studies with patients suffering from this condition. The research design was guided by the researchers of this study and PHC professionals. The list of topics was based on previously published studies [12, 17, 24, 41, 48, 51, 72]. Table 2 shows the list of topics and final questions that were used by the interviewers to guide the interventions. Firstly, the individual interventions were carried out and, secondly, the focus groups were carried out, to note the interactions between the participants, the differences in opinion, the debates, and the dynamics that arose.

**Table 2:** Topic list and questions for patients

Topic list	Questions for patients
Before the interview	1. Greetings, words of thanks, and introduction of the interviewer and observer 2. General information about the topic to be discussed and the objective of the session 3. Explanation of ethical aspects (confidentiality, informed consent, and permission to record) 4. Explanation of the interview dynamics (We will ask some questions to find out about your experiences. We are interested in your opinions. Before we continue, do you have any questions or any doubts? Do you agree to participate?)
Emotional well-being	How do you believe your ongoing symptoms have affected you on an emotional level?
Social support networks	Do you consider yourself to have a social network, such as family and friends, to support you through your ongoing symptoms? How do you consider your social network to be?
Experiences of discrimination and perceived social stigma	Do you believe you have faced discrimination due to having Long COVID? Is there a certain stigma surrounding it?

The objectives of the study were indirectly addressed and the questions asked about the topics were answered in an open manner. The interviewers and/or moderators were introduced to the participants as health professionals and researchers. Specifically, they were two psychologists who were members of the research group and they assumed a minimal role of merely orientating the interviews and focus groups and limited their interventions to addressing the topics in the script. The environment for data collection where the interviews and focus groups took place was a meeting room in a health center. Only those participating in the interviews were present in order to ensure the confidentiality of the responses. The in-depth interviews lasted between 20 and 60 minutes and the discussion groups lasted between 40 and 75 minutes. All the sessions were digitally audio-recorded and transcriptions were made in order to obtain the final set of qualitative data for analysis. None of the interviews were repeated. In this way, as the same interviewers were used throughout the study, they were the ones who perceived that information saturation had already been reached in the focus groups as well as individual interviews.

In order to assess the scope of the discourse, an inductive thematic content analysis was carried out in pairs so as to explore, develop, and define the emergent categories of analysis that derived from the individual interview and group data [73]. This analysis was carried out by two researchers independently, although both used the topic list from which the categories emerged as a guide. There were no discrepancies, except for the idea of abrupt-appearing depression, which was consensual, as shown in the results. Subsequently, the categories that emerged were coded from the list of topics, based on previously published studies [12, 17, 24, 41, 48, 51, 72]. The analysis as a whole was carried out iteratively using the NVivo software, as agreed between the two researchers, and the interpretations of the data were discussed with the interviewers and participants to obtain their consent [74]. In this way, a methodological triangulation was carried out among participants, interviewers, and researchers who participated in the analysis of the results, resulting in greater consistency and rigor and ensuring a correct interpretation of the discourse.

## Results

**Fig. 1:**
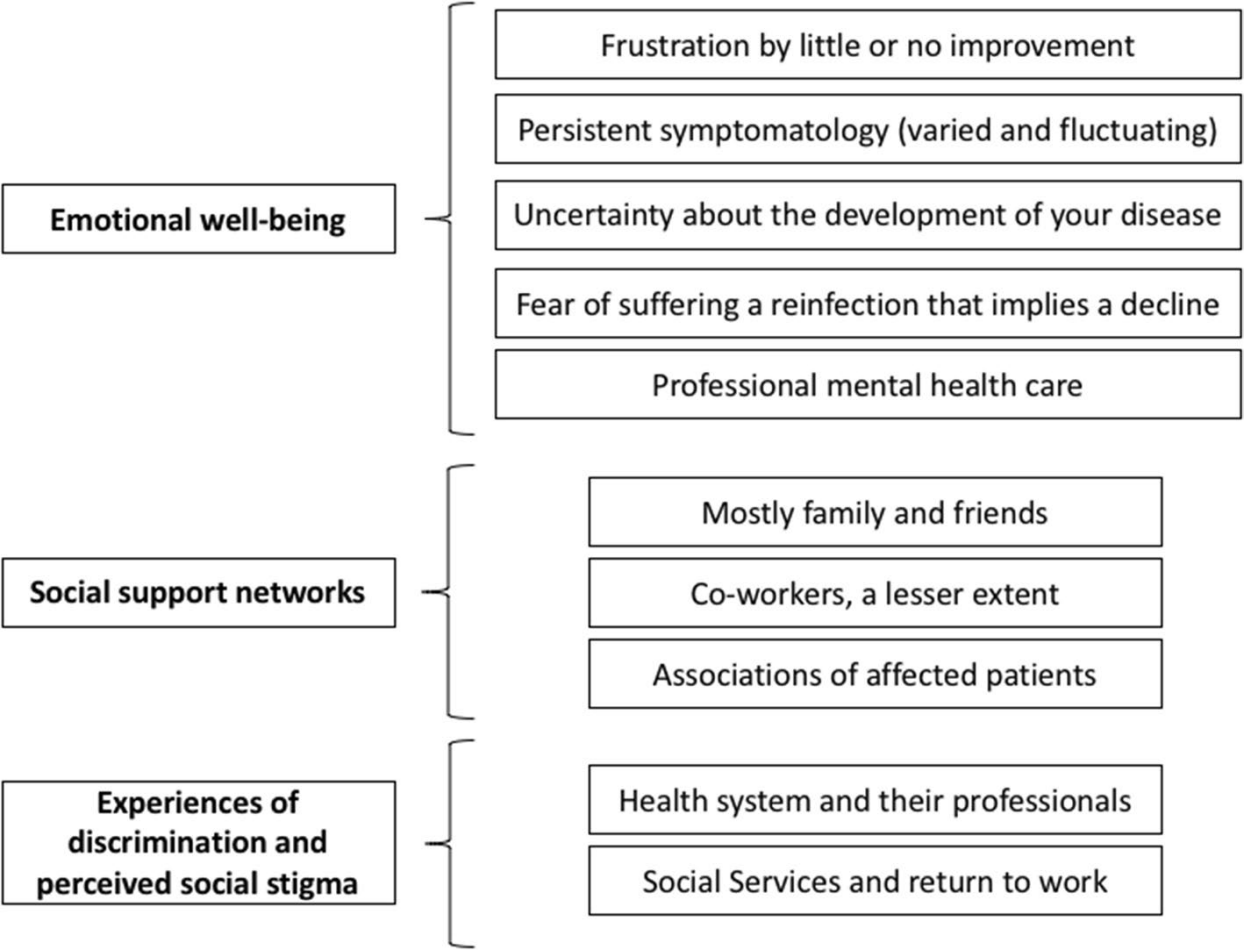
Graphic representation of the central aspects of the results

As shown in Fig. 1, a total of 10 categories were obtained, which after being analyzed, were unified and grouped into 3 main themes: 1) Emotional well-being; 2) Social support networks; and 3) Experiences of discrimination and perceived social stigma.

### Emotional well-being

Very low moods are common among participants with Long COVID due to their symptoms and the limitations they face in their day-to-day lives that appear fairly suddenly and have been ongoing for many months. Patients report that they suffer from sudden episodes of sadness. In the interviews and focus groups, patients mentioned that they feel that having Long COVID is a battle for their bodies and they find it difficult to regain their previous life: they feel that they have suddenly aged and have to mourn the life they have lost. In addition, they are aware that depression and their state of mind make it difficult for them to do rehabilitation exercises and try to resume their previous social life. Suicidal ideas were also mentioned by several participants.

Well, I’ve been through a battle, this is like going through a battle. 1 day you have your life, and the next day it has been taken from you. (Female, 50 years old, 10 months with Long COVID).

I am at my best at home and locked up because I am not the same person I was, and I myself see that I am being consumed. (Male, 62 years old, 20 months with Long COVID).

I’ve been very down for a month and I don’t even feel like doing the respiratory rehabilitation exercises. I [have become] the woman in pajamas. I don’t feel like doing anything. (Female, 39 years old, 12 months with Long COVID).

I also thought about dying... you start to think that you do not want to live like this… (Female, 43 years old, 20 months with Long COVID).

Exactly, I asked myself why I should want to live like this... (Male, 49 years old, 9 months with Long COVID).

They report anguish and anxiety about the future, not only due to how their persistent symptoms could evolve but also due to the fear of possible reinfections and relapses. A fear of reinfection is apparent in the interviews and focus groups. Reinfection could lead to a relapse, and further impact their lives as well as the lives of their relatives and cohabitants.

Then there’s the whole psychological issue, the [fear of] crowds… (Male, 43 years old, 8 months with Long COVID).

For us, catching it is no longer the problem. It’s the fear of what comes after that…. for me catching it again is unthinkable, I don’t know how I would deal with it. So, socially things are very difficult for me... and I also control my environment... (Female, 59 years old, 14 months with Long COVID).

Anguish and anxiety are also reflected in the interviews and focus groups in relation to the fear of having to return to work as they hold the belief that their state of health will not allow them to do their job correctly.

I am very concerned about the future... we should have support for readjusting to work when we return because I struggle to take my briefcase and run to a patient’s emergency room because I am drowning [in work] and cannot do my job at the same pace as before... Now I have somewhat improved my concentration but I have a lot of responsibility. A lot. So, of course, if I make a mistake or commit negligence… (Female, 59 years old, 14 months with Long COVID).

I feel more depressed, and I try to be cheerful, but I wake up at four o’clock in the morning and it’s hard for me to fall asleep. I’m worried, thinking about how this is going to end... Also, I have the issue of work, I am self-employed and have to work... (Male, 56 years old, 16 months with Long COVID).

Additionally, anger and frustration are also mentioned by the participants in response to their health status not having improved for months or still not having medical answers, etc.

At home, we spend the day arguing. Now we argue starting first thing in the morning and really, the most notable thing is that you don’t see a way out. (Male, 40 years old, 11 months with Long COVID).

Many of the participants report that they have sought help from a mental health professional such as a psychiatrist, psychologist, or therapist. All those who have been to a mental health professional consider it to have been a very useful service that has helped them to cope with their situation. There is also a participant who talks about using her faith as a method to cope with the situation. Moreover, many participants have confirmed that the search for information has also been a means of trying to minimize fear, but they recognize that thinking about the uncertain evolution of the illness generates more anxiety. In addition, due to their desperation, some participants have tried to find a solution based on unreliable information. For example, using homeopathy or methods lacking scientific evidence. Therefore, it seems that refraining from seeking information leads to patients coping better.

What worked very well for me was going to a psychiatrist that the medical association gave us. I started to feel better. (Female, 59 years old, 14 months with Long COVID).

Psychologically, it has not affected me much. Perhaps it is due to my faith. I can say that it is thanks to God that I am here to tell the tale. (Female, 51 years old, 19 months with Long COVID).

I used to look for information online but, in the end, I decided that I am not going to look at anything apart from what the doctors tell me to. And now I’m happier. (Male, 60 years, 11 months with Long COVID).

### Social support networks

Most participants report that they have received social support from family and close friends but also think that in social circles that are not as close, such as co-workers, there is less support. They also believe that ignorance about the disease has influenced the support they have received. Besides, many of the patients think that despite the willingness to help, it is not possible for others to understand the symptoms of the disease if they have not experienced it firsthand. Obviously, there is less support if there are COVID-19 deniers in the social network. Finally, the social support they have received from members of the Association of Long COVID Patients, which was created by the patients themselves, has been considered to be very relevant and useful.

Yes, yes, I do. My friends, well, the truth is that, yes, they are my lifelong friends... as well as my daughter, my siblings, all of them, yes. (Female, 63 years old, 20 months with Long COVID).

Yes, I think they try, but no one really understands you unless they have had it. Then, it takes a long time. It is long for me, for all of us. But I do have their support... (Female, 44 years old, 12 months with Long COVID).

I am a nurse, and there was no acknowledgment from my colleagues. Everyone thought I had anxiety when I had tachycardia... I asked for help and did not receive it. It was very hard. I don’t know if it was the physical pain caused by the symptoms that was more difficult, or if it was the lack of understanding from my colleagues... (Female, 50 years old, 18 months with Long COVID).

When news about this began to appear, it was comforting because people then called you and said: “Hey, I’ve heard on the radio that there are more people like you.” And it turns out I wasn’t making this up. For me, that was wonderful. (Female, 64 years old, 20 months with Long COVID).

My daughter, for example, does not believe in COVID and my friends are not vaccinated and do not believe in it. I tell them that I have long COVID but they tell me that I’m making things up in my head, and that makes you feel alone. (Female, 47 years old, 12 months with Long COVID).

### Experiences of discrimination and perceived social stigma

In general, the participants affirm that they have not suffered discrimination from their social circle, including family, friends, and, to a less extent, from co-workers. It does appear in the discourse that most of the participants have suffered experiences of discrimination by health care workers and the social and health system prior to the long COVID disease being recognized. Participants attributed this to the initial lack of knowledge of what it meant to suffer from long COVID and the fear of becoming infected, which worsened throughout the first months and years of the pandemic.

Specifically, it happened to me at the hospital 1 day when I had an appointment with the pulmonologist. I was in the waiting room; I was wearing my mask and I started coughing. Well... I felt like I was a leper... (Female, 51 years old, 19 months with Long COVID).

Yes, there is or was some rejection. In the beginning, when I said that I had long COVID, which was not yet very well known, I was told “Get out of here, you are going to infect us”, and that was from a doctor! They told me that I couldn’t be there and that I should have stayed at home. Stigma doesn’t come directly from people, but rather from fear and a lack of knowledge. More than telling these people you have to… no, it’s the lack of knowledge like what I said at the beginning about doctors, that I had to go. They tell you that this is all psychological, you are somatizing, go to a mental health clinic… but well, fortunately, I think people are getting to know about it more and more. (Male, 44 years old, 12 months with Long COVID).

Long COVID patients acknowledge that PHC professionals have taken an interest in them and have carried out regular follow-ups. However, they have identified greater discrimination on the part of specialist doctors, but they also recognize that there is not enough knowledge, nor action and treatment guidelines for their adequate health care. In general, participants claim that the blame for not knowing how to manage their persistent symptoms belongs to the health care system and not its professionals. Participants who are also health professionals are more critical of their fellow professionals. While they recognize that they are stressed and have limited knowledge and means, they should show more empathy.

The professionals do not believe you and come up with another problem [to explain symptoms], or tell you directly that they do not know what is really best [for you]. Normally, what they do is treat it as a mental condition and it is not... (Male, 43 years old, 8 months with Long COVID).

But I know that I can’t blame the doctors, I’m not going to blame the only ones who can fix it, but those above. What I would like to note is that we must fight for the medical establishment to take us seriously and that my GP goes out of her way for me. (Female, 59 years old, 14 months with Long COVID).

Well, yes, I am angry, not only with the health care system but also with professionals because I have told them: “the situation is stressing you out but we are sick and isolated at home, and you are not paying any damn attention.” The way they are acting is an embarrassment. (Female, 50 years old, 18 months with Long COVID).

Regarding the social security system, they are also very critical and feel discriminated against and misunderstood since most of them are being discharged a year after their sick leave began, and are not well enough to carry out their previous profession. They also criticize the review process and medical tribunals, etc., in which they feel that they are questioned regardless of the medical evidence they present. The participants who are health professionals also criticize the lack of recognition of COVID as an occupational disease when the infection was spread in the workplace.

There is no understanding at an institutional level. They do not help you, quite the contrary. That is what has seemed the hardest to me lately because they discharged me completely without even looking at me and without knowing how you really are, even though you have provided a thousand detailed reports, then that overwhelms you because if you can’t cope with normal daily life, how are you going to consider going back to work? ... They think you’re fine and that you can lead the life that you’re used to, and therefore they don’t help you. (Female, 44 years old, 13 months with Long COVID).

It’s a very unpleasant feeling when you go for the INSS (Spanish National Institute of Social Services) review because you’re treated like an animal. They put me on a chair, glued to a closet, and I had to leave everything on the floor. I was at the exit door next to the closet, in a cubicle as if I was - I don’t know what... it’s outrageous, outrageous. And you’re always trying to show... You feel bad when you go in and worse when you come out. (Female, 59 years old, 14 months with Long COVID).

## Discussion

This qualitative study has collected the self-perceived mental impact of patients with a Long COVID diagnosis. All participants have identified a reduction in their emotional well-being as well as an impairment in their general mental health due to the disease and its exhausting impact on many vital aspects. Elements with the potential to worsen their emotional state have been identified, such as the symptoms themselves, uncertainty about their evolution, and fear of suffering reinfection or discrimination from the health system, among others. In addition, it has been identified how they perceive social support from family and friends, and the positive impact of contact with mental health professionals. These data could serve as support for future research as well as indications for PHC professionals.

The qualitative methodology is an adequate method to obtain novel information of high value, as is the case of the experiences and subsequent sequelae of people infected with COVID-19, as well as the interactions of these patients with their community, health care system, and society [75, 76]. Research using qualitative methodology with Long COVID patients is still scarce, although it is possibly the most common in the study of this group of patients. Some existing articles, based on qualitative methodology, have delved into the emotional well-being of patients with Long COVID [46–51]. It is worth mentioning the study by Burton et al., (2021), which was able to identify factors such as persistent symptoms, lack of treatment, and uncertainty of evolution, which affect the mental health of patients. These results are in line with our findings, as will be detailed later [49]. Unlike other studies, the participants in this research have an average evolution time of 15 months from their initial COVID-19 infection, so their contributions may reflect a broader view of the evolution of the disease, as well as different experiences from a perspective of assimilation and great effort.

The approach and results obtained in this study are in line with the PERMA well-being theory, which states that adapting to living with a disabling or chronic illness has the potential to affect multiple components of well-being. It is also related to a reduced function of the individual regarding less social participation, which can lead to greater social isolation and negatively influence mental health and, in turn, be associated with a higher risk of developing mental pathologies [77, 78].

This study has delved into the emotional impact experienced by patients with Long COVID. Our participants emphasize a before and after in their lives and an irreparable change, referring to the adaptation process they have needed and continue to need to undergo for their new lives. It is not new that chronic diseases can be disruptive events in the lives of those affected [79]. Several studies describe this reality in relation to COVID-19, through ideas such as losing their sense of self, a significant impact on identity, and the separation between a pre-COVID life and a post-COVID life [51, 80–82]. Similar experiences have already been described by Charmaz (1983), who delved into the suffering of patients with chronic diseases caused by physical symptoms and psychological distress, in addition to daily limitations or social isolation [18, 83]. Ladds et al. (2020) describe the disease as “terrifying, confusing, and debilitating”, based on the severity of its symptoms and several aspects reported by affected patients, such as the lack of medical knowledge, uncertain prognosis, and a stagnant evolution with no clear prospects for recovery [18]. In this sense, the participants in this study have established a direct relationship between persistent symptomatology and an anxious-deprived state, in addition to other aspects such as uncertainty. Reinforcing this idea, the previous bibliography affirms that fewer persistent symptoms are related to greater life satisfaction [72]. The Burton et al.’s study (2022) also states that post-COVID patients with ongoing symptoms have experienced mental health effects due to the symptoms themselves, the impact on their quality of life, the lack of care and health services, and the uncertainty of the trajectories of their disease, among other examples [49]. The negative mental effects can be related to physical symptoms, considering that a bidirectional relationship can be established between these variables: physical symptoms lead to poor mental health and a greater mental load can aggravate the perception of the physical symptomatology [35]. In addition, one of the keys to understanding the negative impact on the emotional well-being of these patients would not only be in the symptomatology itself (biological character) but also in the changes to patients’ quality of life and routines (social character) [48]. Delving deeper into this idea, our results have related different negative sensations to their direct causes, according to the subjectivity of the patients, such as uncertainty due to the unknown development of their disease and frustration due to their lack of improvement and the lack of treatment and medical care. Coinciding with these findings, various studies have identified multiple causes that can generate concern, frustration, confusion, and anxiety in this group of patients, including: the lack of information and knowledge about the disease and its causes; ignorance of its evolution and the lack of treatment; the health care received; and the functioning of the health care system during the COVID-19 pandemic [18, 50, 80].

Regarding social support, most of the participants in this study have recognized family and friends as one of their main forms of support. Some studies confirm that this group of patients seek acceptance and understanding from those close to them, including family and friends, and also from health professionals [51]. In addition, other studies show how a multitude of family and friends were not only a source of emotional support but also offered their help with household chores or basic activities, although sometimes these actions turned out to be accompanied by misunderstanding and ignorance about the disease [48]. This last idea was reflected in our results, given that many participants say they feel understood only by other patients who experience the disease firsthand. In this sense, Macpherson et al. (2022) add that the closest relatives of the patients also require support, based on the need to understand the disease, which was recently cited by Ireson et al. (2022) [48, 51]. In short, family and friends are important companions who positively influenced the emotional well-being of patients, generating peace of mind in the face of various adversities, although some of them require professional action guidelines [81]. This study has also identified other sources of support, such as patients seeking alternative therapy or treatments, and professional help and mutual help groups, such as the Association of Long COVID Patients of Aragon, which has played a fundamental role in patients’ attitudes, as reported by some of our participants [19]. As far as we know, no research has yet identified those elements with the potential to improve the emotional well-being of patients with Long COVID, and, as such, there should be an incentive to continue with this research.

Regarding the stigma generated, our study has identified two main sources of discrimination: the health care system and social services in relation to the workplace. Like in other studies [18, 80], a stigma generated by the general population, based on mistaken beliefs and ignorance of the disease, has been identified. In relation to the health system, patients with Long COVID claim to have encountered many difficulties in being treated by the relevant medical services, including mental health services [18]. This study has not been the only one to identify that, when patients try to receive optimal care, they are met with barriers, such as a lack of scientific knowledge about the disease, which has led some health professionals to question the veracity of the patient’s symptoms or has resulted in them associating the symptoms with psychiatric origins without carrying out previous tests [48]. Both a lack of knowledge and discriminatory treatment in health care settings have contributed to the emotional distress of many patients [50, 51, 82, 84]. As pointed out by Hadler (1996), “If you have to prove that you are sick, you cannot get better” [85]. Very similar experiences have been reported by fibromyalgia patients, who, at the beginning of the disease, had to fight for its diagnostic recognition, due to the refusal of health professionals to accept it [86]. Moreover, in relation to the workplace and, specifically, the process of returning to work is one of the issues that has caused the most anxiety and concern [81]. Our participants verify that there is a relationship between the inability to return to work and poor mental health, which is one of the biggest concerns for patients. This concern is not only due to their poor physical condition but also due to their cognitive and mental impact [18]. Regarding the process of being assessed by a medical committee to return to work, there are no other studies that have further explored the experiences of patients when describing this step and the misunderstanding generated when discharged from work. For many of our participants, the treatment they have received has been inhumane. Several of them have requested protection measures and investigations to carry out an adaptation of this process. These discriminatory experiences must be taken into account because they may have a negative impact on the health of those affected, thus generating health inequalities in stigmatized patients with Long COVID [87].

The results of this study suggest the need to address the mental symptoms that Long COVID patients have developed from a clinical perspective. Our patients have perceived improvements in their well-being after being treated by mental health professionals. In fact, some health care centers have implemented low-threshold therapies or counseling for the general population affected by COVID-19 to promote emotional support in the early stages of the disease [88]. Thus, the implementation of brief questionnaires aimed at identifying the psychological needs of patients with Long COVID is proposed to encourage referrals to specialized health professionals who support these patients in their mental recovery process alongside their physical rehabilitation [36]. As an alternative to the lack of specialized or complementary mental health services, Gómez-Conesa (2021) proposes approaching the different mental problems of patients with Long COVID using rehabilitation physiotherapy [89]. Nonetheless, there is still a lack of attention with regard to Long COVID and the prevalent mental health problems experienced by the patients [90]. Indeed, the multidisciplinary approach required in the management of this disease may become one of the greatest challenges for the health and social security system in the coming years [91]. It is necessary to not only implement purely clinical approach strategies, since this group of patients also narrates how their relationships with their community and society at the time have been affected by their illness, developing a tendency toward social isolation. For this reason, community reintegration must also be considered from a clinical point of view. Some studies refer to the effectiveness of the social prescription methodology in the context of the COVID-19 pandemic. This method allows GPs and other PHC professionals to suggest social and non-clinical activities to do in the community to their patients, thus caring for their physical health, as well as helping create a feeling of belonging within the community [92]. Social prescription was an important source of support for many people, especially for vulnerable groups, in the face of the interruption of various services due to the pandemic [93–95]. In addition, social prescription has the potential to improve the emotional well-being of the population [96], as shown in this study with community participation in the previously-mentioned Association. Therefore, given the results obtained and the lack of available treatments, this technique could be a useful tool for PHC professionals and improve the emotional well-being of the patients.

Regarding the strengths of this study, we believe that our data are representative of our patient population as it includes a wide range of sociodemographic profiles. Additionally, a large part of the sample was diagnosed over a year ago, and consequently, they have shared stories and experiences that are very rich in content from an evolutionary perspective, along with examples of the unawareness of those diagnosed most recently. In addition, the choice of a qualitative methodology has made it possible to actively evaluate Long COVID, its different areas of involvement, and the emotional impact in relation to important aspects of the participants’ lives. A limitation of this study was that we did not assess poor psychiatric health prior to the COVID-19 infection and, as such, the results cannot express the prevalence of psychiatric illness before and after infection. In addition, by not collecting the experiences of mental health professionals, the subjective discourse of the participants makes it impossible to explain the complex relationship between pre-existing psychiatric illness and the burden of Long COVID on their current mental health.

## Conclusions

Patients with Long COVID experience various factors that negatively affect their mental health and emotional well-being. Their personal testimonies are essential to understand and treat the disease with a comprehensive approach, since the biomedical model based on objective indicators is not consistent, which generates a stigma within health care. For this reason, health and social services must implement and strengthen access routes, care, guidance, and new programs for professionals addressing both the physical and mental health of patients with Long COVID.

## Data Availability

The datasets used and/or analyzed during the current study are available from the corresponding author upon reasonable request.
